# UV Hyperspectral Imaging with Xenon and Deuterium Light Sources: Integrating PCA and Neural Networks for Analysis of Different Raw Cotton Types

**DOI:** 10.3390/jimaging10120310

**Published:** 2024-12-05

**Authors:** Mohammad Al Ktash, Mona Knoblich, Max Eberle, Frank Wackenhut, Marc Brecht

**Affiliations:** 1 Process Analysis and Technology PA & T, Reutlingen University, Alteburgstraße 150, 72762 Reutlingen, Germany; mona.knoblich@reutlingen-university.de (M.K.); max.eberle@reutlingen-university.de (M.E.); frank.wackenhut@reutlingen-university.de (F.W.); 2 Institute of Physical and Theoretical Chemistry, Eberhard Karls University Tübingen, Auf der Morgenstelle 18, 72076 Tübingen, Germany

**Keywords:** UV hyperspectral imaging, pushbroom, cotton, principal component analysis, PCA, quadratic discriminant analysis, QDA, PCA-QDA, xenon arc, deuterium, neural network, deep learning

## Abstract

Ultraviolet (UV) hyperspectral imaging shows significant promise for the classification and quality assessment of raw cotton, a key material in the textile industry. This study evaluates the efficacy of UV hyperspectral imaging (225–408 nm) using two different light sources: xenon arc (XBO) and deuterium lamps, in comparison to NIR hyperspectral imaging. The aim is to determine which light source provides better differentiation between cotton types in UV hyperspectral imaging, as each interacts differently with the materials, potentially affecting imaging quality and classification accuracy. Principal component analysis (PCA) and Quadratic Discriminant Analysis (QDA) were employed to differentiate between various cotton types and hemp plant. PCA for the XBO illumination revealed that the first three principal components (PCs) accounted for 94.8% of the total variance: PC1 (78.4%) and PC2 (11.6%) clustered the samples into four main groups—hemp (HP), recycled cotton (RcC), and organic cotton (OC) from the other cotton samples—while PC3 (6%) further separated RcC. When using the deuterium light source, the first three PCs explained 89.4% of the variance, effectively distinguishing sample types such as HP, RcC, and OC from the remaining samples, with PC3 clearly separating RcC. When combining the PCA scores with QDA, the classification accuracy reached 76.1% for the XBO light source and 85.1% for the deuterium light source. Furthermore, a deep learning technique called a fully connected neural network for classification was applied. The classification accuracy for the XBO and deuterium light sources reached 83.6% and 90.1%, respectively. The results highlight the ability of this method to differentiate conventional and organic cotton, as well as hemp, and to identify distinct types of recycled cotton, suggesting varying recycling processes and possible common origins with raw cotton. These findings underscore the potential of UV hyperspectral imaging, coupled with chemometric models, as a powerful tool for enhancing cotton classification accuracy in the textile industry.

## 1. Introduction

Sensor technology plays a crucial role in enhancing imaging systems, such as hyperspectral imaging. This technology detects and analyzes specific spectral signatures [1]. Hyperspectral imaging captures a series of images at different wavelengths to generate a comprehensive spectrum of the material. It is rapid, non-destructive, and robust. Hyperspectral imaging, particularly when employed as an on-line monitoring tool, combines spectral and spatial information. This can result in a large amount of spectral data within a short period [2,3]. Consequently, multivariate data analysis methods, such as principal component analysis (PCA), are needed. PCA is a statistical method used for dimensionality reduction while preserving the variance in high-dimensional data. It transforms the original variables into uncorrelated principal components, with loading weights indicating the contribution of each feature. This technique can identify clusters and patterns by plotting samples in PCA space, highlighting the differences among sample groups. The first principal component captures the most variance, which aids in interpreting the results. PCA helps differentiate between sample types based on their characteristics, revealing influential features and enhancing the understanding of the underlying biological or environmental processes that drive the observed variations [4,5]. PCA is essential to reduce data volumes while keeping critical information. It effectively reveals similarities and differences among samples within large data matrices [6,7,8,9]. For the classification and quality assessment of parameters, PCA is often combined with a Quadratic Discriminant Analysis (QDA) model [10]. In recent years, deep learning techniques have emerged as powerful tools for classification tasks, offering improved accuracy and robustness over traditional methods. Neural networks, in particular, enable the modelling of complex relationships within data, making them suitable for various classification and quality assessment applications. This combination is particularly effective in determining material quality and is widely applied across various fields [11,12]. In agriculture, hyperspectral imaging facilitates the monitoring of crop health by identifying biotic stressors and nutrient deficiencies [13], while in medicine, it enhances quality control processes and aids in distinguishing between different drug types and active pharmaceutical ingredients [3]. Additionally, in environmental studies, it plays a critical role in evaluating water quality parameters such as chlorophyll concentrations and pollutant levels [14], and in textiles, it allows for the precise classification of fiber types, thereby ensuring adherence to industry standards [15,16]. Furthermore, waste sorting techniques, such as refurbishing plasterboard waste samples [15], and applying hyperspectral imaging for food quality control in the food industry have shown significant potential [17].

In the textile industry, hyperspectral imaging is increasingly used to evaluate the quality of raw materials, such as cotton [6,18]. Cotton is a crucial raw material in textile manufacturing, accounting for approximately 30% of all fibers used in the textile sector and holding significant economic importance [19]. Therefore, ensuring high-quality cotton is essential for economic stability and serves as a vital income source for certain countries. However, accurately differentiating between various cotton types remains a challenging task [20]. In recent years, the emphasis on using organic cotton produced under fair conditions has grown significantly due to its environmentally friendly nature, as it is cultivated without synthetic chemicals, unlike conventionally produced cotton [21,22]. Additionally, compliance with recycling quotas has become increasingly important, as recycled cotton, derived from post-consumer or post-industrial waste, reduces the need for new raw materials [23]. Consequently, identifying the origin of cotton and understanding the distinctions between organic, conventional, and recycled cotton is crucial for making informed purchasing decisions and supporting sustainable practices [24].

Numerous methods have been developed for the identification and classification of different cotton types. Traditional techniques, such as thermogravimetric analysis and high-performance liquid chromatography (HPLC), have been utilized; however, these approaches exhibit certain limitations [23,25,26]. Other approaches are based on various spectroscopic methods. In particular, the visible (Vis) spectral range yields limited information, largely due to raw cotton’s reflective or transparent properties and residues [25,27]. Conversely, the near-infrared (NIR) region offers valuable insights through characteristic molecular vibrations, such as those of CH_n_ and OH groups, which are prevalent in cotton. Despite this advantage, the NIR range demonstrates limited sensitivity to minor variations or contaminants, complicating the detection process [27,28,29,30,31,32]. Consequently, numerous studies in the NIR region have integrated spectroscopy, hyperspectral imaging, and chemometric modelling to enhance detection capabilities [29,33].

Nevertheless, the application of hyperspectral imaging in the NIR range for cotton classification has encountered limitations. To address this, the present study employs hyperspectral imaging in the ultraviolet (UV) range (225–408 nm). This approach is contrasted with the work of Al Ktash et al. [28], which explored various cotton samples using a benchtop spectrometer across the UV–Vis/NIR range, as well as NIR hyperspectral imaging. Their findings indicated that significant spectral features are present in both the UV and NIR ranges. However, while NIR hyperspectral imaging was available for sample analysis at the time of their study, UV hyperspectral imaging was not yet commercially accessible. Consequently, this study advances the field by developing and utilizing UV hyperspectral imaging with two different illumination sources: xenon lamps (XBO) and an SL3 deuterium lamp, for the differentiation of distinct cotton sample types.

Using XBO and deuterium light sources is important for optimizing applications in spectroscopy and analytical chemistry due to their distinct spectral ranges and stability characteristics. XBO lamps provide a broad spectrum, from UV to visible light, making them suitable for various applications, while deuterium lamps are specialized for the UV range and are ideal for specific measurements requiring precise UV absorbance. Additionally, the intensity of XBO lamps in the deep UV regions is weaker compared to that of deuterium lamps [16,34,35,36,37]. A chemometric model, based on PCA, was developed and successfully employed, enabling effective differentiation among various cotton types and hemp samples within the dataset. Furthermore, to ensure the effectiveness of the study, a neural network deep learning technique was applied. This involve developing a fully connected neural network.

## 2. Materials and Methods

### 2.1. Samples

**Figure 1:**
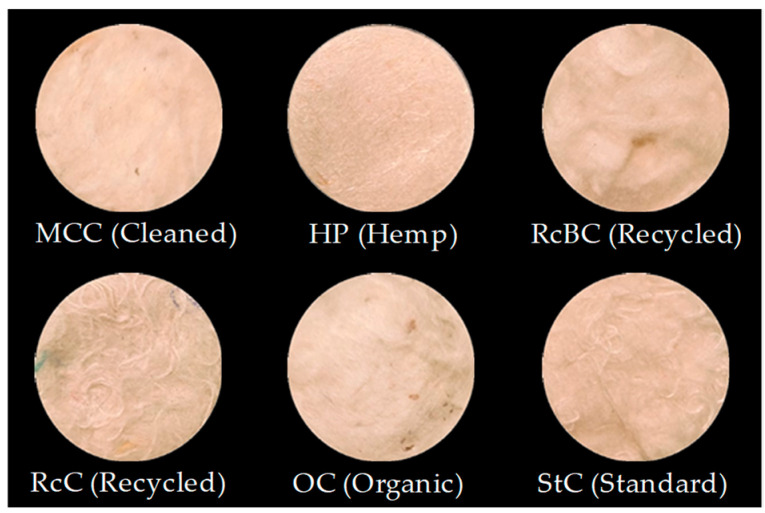
Visual representation of the pressed disc-shaped samples. The samples were mechanically cleaned cotton (MCC), raw hemp plant (HP), recycled organic bright cotton (RcBC), recycled cotton (RcC), organic raw material cotton (OC), and standard raw material cotton (StC).

In Figure 1, six different types of raw samples, including five cotton varieties and one hemp sample, are demonstrated. The samples comprised organic raw material cotton (OC), raw hemp plant from China (HP), recycled cotton (RcC), standard raw material cotton (StC), recycled organic bright cotton (RcBC), and mechanically cleaned cotton (MCC). The MCC was supplied by ICA Bremen GmbH (Bremen, Germany). For each cotton type, three samples were randomly selected from the bulk material, with each sample weighing 1 g. In total, 18 samples were investigated. The samples were compressed into a disc shape using a hydraulic press (PerkinElmer, Inc., Waltham, MA, USA) at a pressure of ten tons for two min to ensure uniform physical properties across all samples. The hydraulic press was thoroughly cleaned between pressing each sample to prevent cross-contamination. The pressed samples were then placed in a sample holder to maintain consistent morphology during the hyperspectral imaging scan.

### 2.2. UV Hyperspectral Imaging Setup

The UV hyperspectral imaging setup employed a pushbroom technique, utilizing two different illumination sources: two xenon lamps (XBO, 14 V, 75 W, OSRAM, München, Germany) and an SL3 deuterium lamp (24 V, 65.04 W; StellarNet Inc., Tampa, FL, USA). The imaging system consisted of a back-illuminated Charge-Coupled Device (CCD) camera (Apogee Alta F47: Compact, inno-spec GmbH, Nürnberg, Germany) connected to a spectrograph (RS 50-1938, inno-spec GmbH, Nürnberg, Germany) with a slit width of 30 µm. The CCD camera offered a resolution of 1024 × 1024 pixels (spatial × spectral), with a pixel size of 13 µm × 13 µm and an integration time of 300 ms. The samples were then positioned on a black conveyor belt (700 mm × 215 mm × 60 mm, Dobot Magician, Shenzhen Yuejiang Technology Co., Ltd., Shenzhen, China), which moved at a constant speed of 0.15 mm/s during the imaging process. The XBO lamps were used in combination with a polytetrafluoroethylene (PTFE) (Sphereoptics GmbH, Herrsching, Germany) tunnel to ensure homogeneous illumination. The conveyor belt was fully enclosed within the PTFE tunnel for the XBO setup. The deuterium lamp provided direct, focused illumination without the use of a tunnel.

### 2.3. Data Processing

Multivariate data analysis (MVA) was performed using a self-written software package, HydraX (MATLAB 9.2.0, Mathworks, MA, USA). The UV spectral data were binned by a factor of two and underwent preprocessing steps that included linear baseline correction, Savitzky–Golay smoothing (21 points, symmetric, 2nd polynomial order), and maximum normalization (max. intensity = 1). PCA models were developed using mean centering, full cross-validation, and the Singular Value Decomposition (SVD) algorithm to distinguish between the various cotton types and hemp samples. Additionally, the PCA models were combined with QDA using five principal components (PCs) to classify, assess, and quantify the models. A neural network was built and evaluated using Python (3.10, Python Software Foundation), utilizing the packages Tensorflow (2.17.0) and Scikit-Learn (1.3.2). For data preprocessing, the Python packages Scipy (1.10.1) and Numpy (1.26.4) were used. The UV spectral data were binned by a factor of two and underwent preprocessing steps, including linear baseline correction and Savitzky–Golay smoothing (21 points, symmetric, 2nd polynomial order) for the data generated using the XBO lamp.

## 3. Results

### 3.1. Spectra and Statistical Models

**Figure 2:**
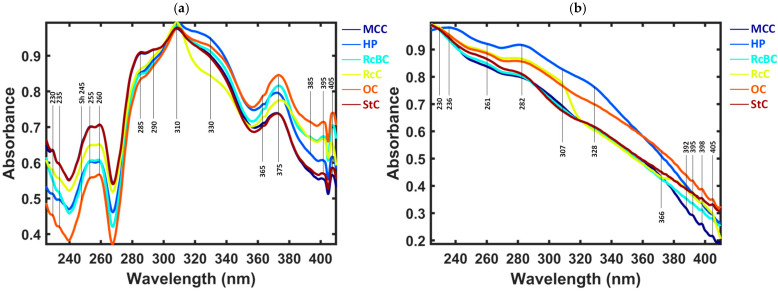
Averaged spectra recorded by UV hyperspectral imaging of raw cotton samples and hemp with (**a**) XBO as a light source and (**b**) deuterium as a light source. The samples were mechanically cleaned cotton (MCC), raw hemp plant (HP), recycled organic bright cotton (RcBC), recycled cotton (RcC), organic raw material cotton (OC), and standard raw material cotton (StC).

Figure 2 shows the averaged spectra produced by the hyperspectral imaging prototype, incorporating two distinct light sources: an XBO lamp and a deuterium lamp. The samples were measured in the reflectance mode in the UV range (225–408 nm) and then averaged. A total of 6120 spectra were collected using the XBO light source, while 5674 spectra were acquired with the deuterium light source in the hyperspectral imaging prototype. A linear baseline correction, smoothing, and maximum normalization were applied to the raw spectra. Distinct spectral features were observed across various regions in all spectra by using XBO as a light source, as shown in Figure 2a. The most prominent bands were observed around < 250 nm, 260 nm, 280 nm, 310 nm, and 375 nm, corresponding to the presence of proteins (specifically, aromatic amino acids such as tryptophan, tyrosine, and phenylalanine) [38,39], DNA [39], pectin [40], and lignin [41]. Overall, the spectral features followed a similar shape. On closer inspection, there were wavelength ranges that differed. The spectra exhibited subtle differences in the deep UV region < 250 nm, with the OC sample displaying the lowest intensity and the StC sample displaying the highest. However, at 400 nm, this pattern was reversed, with the StC and OC samples showing inverted intensity levels. The spectral intensities re-ordered, with the HP sample exhibiting the highest intensity, followed by RcC and RcBC at >310 nm. The intensities of OC and RcC were inverted, and additional samples with varying intensities, such as RcBC, became notable at >310 nm onward. In conclusion, conventional cotton, represented by StC, was distinguishable from organic cotton (OC). Additionally, recycled cotton (RcBC, RcC) was clearly differentiable from raw cotton (OC, StC), and the cotton samples from different plants, such as HP, exhibited distinct differences. The averaged MCC spectrum seemed to be similar to the StC spectrum.

Figure 2b represents the averaged spectra for the different cotton types and hemp using the deuterium lamp in the UV range. The most prominent spectral bands were observed at approximately 230 nm, 236 nm, 261 nm, 282 nm, 307 nm, 328 nm, and 366 nm, with additional smaller spectral features > 390 nm. These bands corresponded to the presence of biomolecules such as proteins, specifically aromatic amino acids like tryptophan, tyrosine, and phenylalanine [38,39], DNA [39], pectin [40], and lignin [41]. The HP sample exhibited the most pronounced spectral features between 230 nm and 360 nm, with higher intensities in this range, while the RcC sample showed significant features from 225 nm to 307 nm, followed by a drop to the lowest intensity at 366 nm. The OC sample displayed the highest intensity at >360 nm. Other samples showed similar intensity patterns around 285 nm, with overlapping spectra between 310 nm and 366 nm, before diverging again at 366 nm. Both the XBO and deuterium lamps revealed characteristic differences in the cotton samples, but the spectral regions and intensity patterns varied between the two light sources. Under XBO illumination, the reordering of spectral intensities > 310 nm distinguished raw hemp (HP), recycled cotton (RcC, RcBC), and organic cotton (OC) from conventional cotton (StC). Meanwhile, the deuterium lamp provided clearer intensity contrasts between 236 nm and 366 nm, especially for the HP and OC samples. In both cases, the recycled and organic cotton could be distinguished based on their spectral features, though the spectral order and intensity patterns differed depending on the light source used.

**Figure 3:**
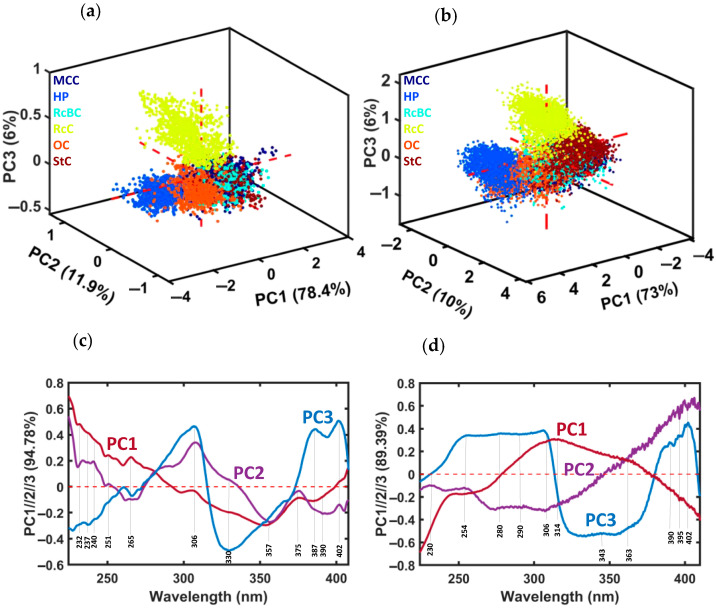
PCA model, calculated using two light sources, XBO and deuterium, with the following scores: (**a**) PC1 (78.4%) vs. PC2 (11.9%) vs. PC3 (6%) using XBO. (**b**) PC1 (73%) vs. PC2 (10%) vs. PC3 (6%) using deuterium. (**c**,**d**) The corresponding loading plots for XBO and deuterium, respectively.

**Figure A1:**
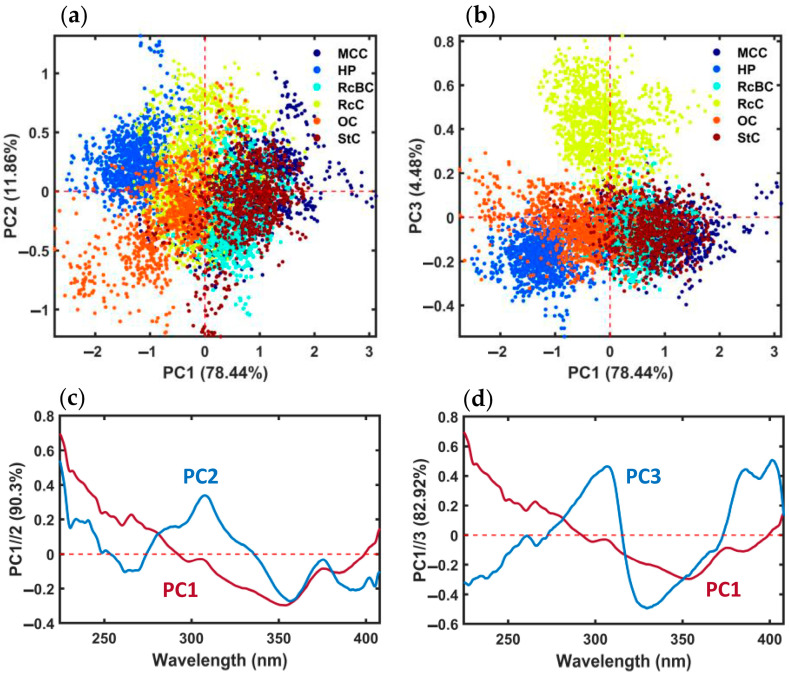
Two-dimensional scores of the PCA model with (**a**) PC1 vs. PC2 and (**b**) PC1 vs. PC3. (**c**,**d**) Corresponding loadings.

**Figure A2:**
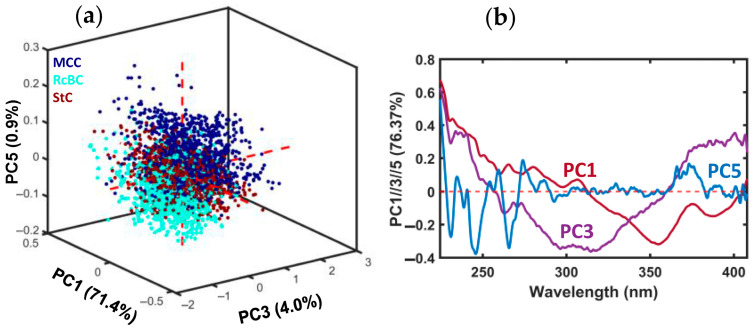
PCA model with MCC, RcBC, and StC; (**a**) scores of PC1 vs. PC2 vs. PC3 and (**b**) corresponding loadings.

**Figure A3:**
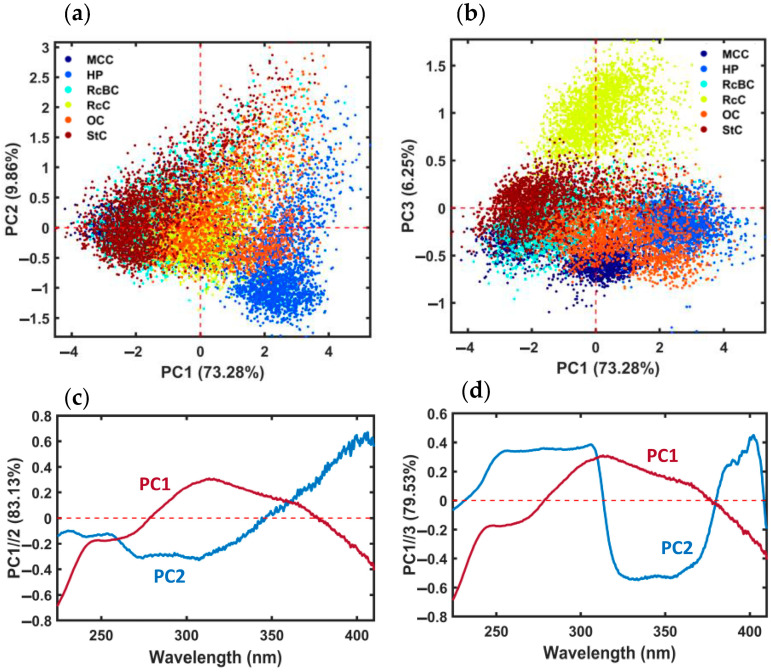
Two-dimensional scores of the PCA model with (**a**) PC1 vs. PC2 and (**b**) PC1 vs. PC3. (**c**,**d**) Corresponding loadings.

**Figure A4:**
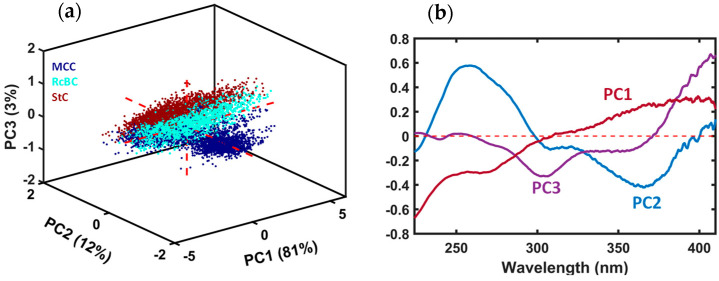
PCA model with MCC, RcCB, and StC; (**a**) scores of PC1 vs. PC2 vs. PC3 and (**b**) corresponding loadings.

Figure 3 presents the PCA model for the different sample types using both lamps. Figure 3a shows the scores plot for the first three PCs, which together explained approximately 94.8% of the total variance when using the XBO lamp. PC1 and PC2 were able to nearly cluster the samples into four main groups, i.e., HP, RcC, OC, and the others (MCC, RcBC, and StC). PC3 clearly separated the RcC sample. PC1 vs. PC2 and PC1 vs. PC3 are shown as 2D scores in Appendix A, Figure A1a,b. The overlap observed among the MCC, RcBC, and StC samples could be attributed to the chemical processes involved during harvesting and subsequent treatments. StC undergoes chemical treatment during cleaning, while MCC and RcBC are exposed to chemicals during recycling. These shared chemical interactions could have likely contributed to the observed spectral overlap among these cotton samples. Moreover, the recycled samples (RcBC and RcC) were clearly distinguishable. This outcome suggests that variations in recycling processes among different companies contributed to the observed difference. To further investigate and highlight the spectral differences, a separate PCA was performed on the MCC, RcBC, and StC cotton samples, resulting in a partial distinction between them, as shown in Appendix A, Figure A2a. Figure 3b shows the scores plot for the first three PCs, which explained approximately 89.4% of the total variance using the deuterium lamp. PC1 and PC2 grouped the samples into four distinct clusters, separating the HP, RcC, and OC samples from the remaining ones (MCC, RcBC, and StC). Additionally, PC3 clearly separated the RcC sample from the others. The 2D scores for PC1 vs. PC2 and PC1 vs. PC3 are shown in Appendix A, Figure A3a,b. These results closely align with the XBO findings shown in Figure 3a, demonstrating strong consistency between the two datasets. Again, a separate PCA was performed on the MCC, RcBC, and StC cotton samples to further investigate and highlight the spectral differences, resulting in a near distinction between them, as shown in Appendix A, Figure A4a.

Figure 3c shows the loading plots using the XBO light source for PC1, PC2, and PC3. The most significant differences among PC1, which occurred at a maximum of 225 nm, were attributed to the presence of DNA [39] and phenylalanine [38,39]. The minimum of 350 nm was associated with chromophores and pectin within the cotton fibers [38,39]. At 300 nm, 305 nm, 330 nm, 375 nm, and 385 nm spectral details appeared. PC2 described the different spectral features observed, with a minimum of 355 nm due to presence chromophores and pectin [38,39]. The maximum between 225–260 nm was related to DNA [39] and phenylalanine [38,39]. A few more details were observed in the wavelength ranges of 270–335 nm and 360–385 nm. The bands around 310 nm and >385 nm exhibited the maximum influence on PC3, reflecting the presence of chromophores and pectin [38,39], with minima at 230 nm and 330 nm corresponding to the presence of chromophores and pectin [38,39], and DNA [39] and phenylalanine [38,39]. Figure 3d shows the loading plots for PC1, PC2, and PC3 using the deuterium light source. The most significant differences in PC1 occurred at a minimum of 225 nm, which was attributed to of DNA [39] and phenylalanine [38,39], and at a maximum of 316 nm, which was due to the presence of chromophores and pectin [38,39]. PC2 described the spectral features with a minimum of <300 nm and a maximum of 400 nm, indicating the presence of DNA [39], phenylalanine [38,39], and pectin [38,39], respectively. Additional details were observed in the wavelength range of 225–254 nm and at 284 nm. The bands from around 255 nm to 307 nm and >390 nm exhibited the maximum influence on PC3, with minima between 323–366 nm. These spectral bands < 250 nm were attributed to the presence of DNA [39] and phenylalanine [38,39], while protein-related features were observed around 280 nm. The spectral bands > 350 nm were associated with chromophores and pectin within the cotton fibers [38,39].

The loadings of the combined PCA of the MCC, RcBC, and StC samples were calculated using the XBO and deuterium light sources, as shown in Appendix A, Figure A2b and Figure A4b, respectively. For the model calculated using the XBO light source, PC1 of the three-sample model followed a pattern similar to that in the previously described XBO PCA of all samples. The bands around 230 nm and 370 nm exhibited the maximum influence on PC3, with a minimum of 315 nm. The main spectral features of PC5, shown in Appendix A, Figure A2b, were located in the wavelength range from 230–270 nm. Similarly, PC1 of the three-sample model showed a pattern consistent with the earlier deuterium PCA for the model using the deuterium light source (Appendix A Figure A4b). The bands around 230 nm and 370 nm exhibited the maximum influence on PC2, with a minimum at 315 nm, while the main spectral features of PC3 were observed in the 230–270 nm wavelength range. In conclusion, conventional cotton, represented by StC, was distinguishable from organic cotton (OC) on PC1. Recycled cotton (RcBC) overlapped with StC and MCC, possibly due to it being recycled from the same conventional origin. RcC was clustered separately from raw cotton (OC and StC) on PC3, and the cotton samples from different plants, such as HP, exhibited distinct differences on PC2.

A comparison of the model results using XBO light and deuterium light showed a close similarity. PC1 was the most different PC due to the similarity of the lamps’ spectral profiles. PC2 and PC3 were comparable for both lamps when PC2’s direction was flipped. However, the model utilizing deuterium light was more effective in distinguishing between the three cotton types (MCC, RcBC, and StC) using the first three PCs, whereas the model with XBO light required five PCs to achieve the same level of separation between the three cotton types.

To validate the robustness of the PCA models (Figure 3), QDAs were conducted using the scores from the first five PCs. The confusion matrices generated utilizing the two light sources, XBO and deuterium, are presented in Table 1 and Table 2, respectively. These matrices provide a detailed overview of the classification performance for the different cotton types and HP samples. The confusion matrix in Table 1 details the classification results for the various cotton types and HP samples, offering a comprehensive evaluation of the model’s classification accuracy [10]. The model achieved an overall accuracy of 76.1% and a sensitivity of 75.9% for the spectra using the XBO light source, while the deuterium light source resulted in an accuracy of 85.1% and a sensitivity of 85.2%. The dark-shaded diagonal elements in Table 1 and Table 2 represent correctly classified samples, while the off-diagonal samples correspond to misclassified spectra.

Table 1 shows that a total of 6120 spectra were collected using the XBO light source, while Table 2 shows that a total of 5674 spectra were acquired with the deuterium light source in the hyperspectral imaging prototype. The results from the hyperspectral imaging using the XBO light source demonstrate that the conventional cotton (StC) spectra were correctly classified as distinct from those of organic cotton (OC). However, there were minor misclassifications: 4 OC spectra were predicted as StC, and 68 StC spectra were predicted as OC. Many RcBC spectra were misclassified as StC, supporting the hypothesis that the RcBC could have been recycled from conventional cotton. The MCC spectra were misclassified as RcBC and StC, which indicates their high similarity to each other. Nearly 100% of the RcC spectra and 96% of the HP spectra were correctly classified and distinguished.

The results from the hyperspectral imaging using the deuterium light source demonstrate that the spectra of conventional cotton (StC) were accurately classified as distinct from those of HP and RcC, with only minor misclassifications: 26 OC spectra were predicted as StC, and 33 StC spectra were predicted as OC. Additionally, 87 RcBC spectra were misclassified as StC, further supporting the hypothesis that the RcBC may have been recycled from conventional cotton. MCC spectra were misclassified as OC, RcBC, and StC, indicating a high similarity among these samples. Nearly 100% of the RcC spectra and 99% of the HP spectra were correctly classified and distinguished. In conclusion, conventional cotton can be distinguished from organic cotton, recycled cotton can be distinguished from raw cotton, and hemp plant can be distinguished from cotton.

**Figure 4:**
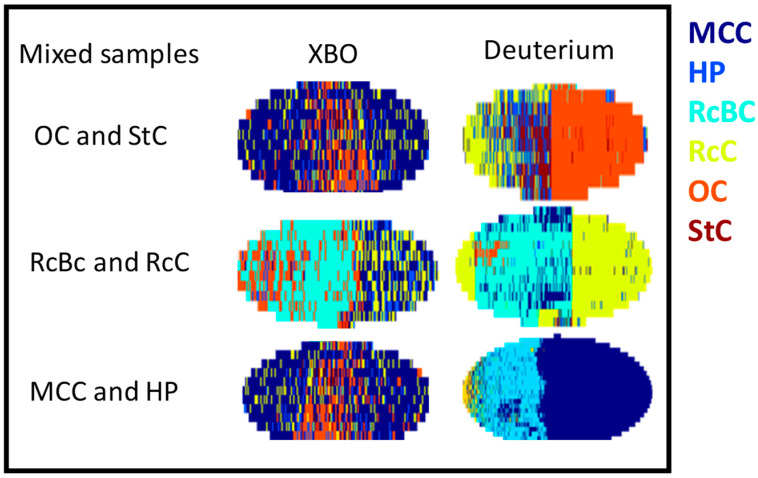
Prediction of mixed cotton and hemp samples using PCA-QDA using XBO (column 1) and deuterium lamps (column 2). First row: mixture of OC (orange) and StC (brown). Second row: mixture of RcBC (light green) and RcC (yellow). Third row: mixture of MCC (dark blue) and HP (light blue).

To further confirm this study’s validity, three mixed samples were prepared, each containing either two primary cotton types or a hemp sample. These mixed samples consist of two halves, where each half represents a sample type. Figure 4 presents these mixed samples and their classification predictions using PCA-QDA under both XBO and deuterium lamp illumination. The sample in the first row was a combination of types OC (orange) and StC (brown). Under XBO lamp illumination, the predictions were less accurate, with OC (orange) being nearly correctly classified, while StC (brown) was misclassified as MCC (dark blue) and RcC (yellow) under the deuterium lamp. This finding supports the hypothesis that the MCC (dark blue) and RcC (yellow) samples may have originated from commercial cotton types. The middle sample included types RcBC (light green) and RcC (yellow). Both lamps accurately classified most RcC (yellow) pixels, although a few were misclassified as OC, with the XBO lamp performing less accurately than the deuterium lamp. The sample in the third row consisted of MCC (dark blue) and HP (light blue), which were clearly distinguishable under the deuterium lamp, while the XBO lamp produced more frequent misclassifications.

These results demonstrate that the deuterium lamp generally provided more accurate classifications across all samples compared to the XBO lamp, especially in distinguishing between MCC and HP and between StC and the other cotton types. This reinforces the utility of deuterium lamp illumination in improving the accuracy of PCA-QDA classification in cotton and hemp mixture analysis.

Furthermore, a deep learning technique was applied. This involved developing a fully connected neural network, with an input layer, an output layer, and three hidden layers, consisting of 128, 64, and 32 nodes, respectively. The hidden layers employed the Rectified Linear Unit (ReLU) activation function to introduce non-linearity by setting all negative values to zero. Dropout layers were implemented after each hidden layer to prevent overfitting. The Adam optimizer was utilized for the training process to minimize the sparse categorical cross-entropy loss, ensuring the model’s accuracy. The softmax activation function was applied in the output layer to convert the final outputs into probabilities.

**Figure A5:**
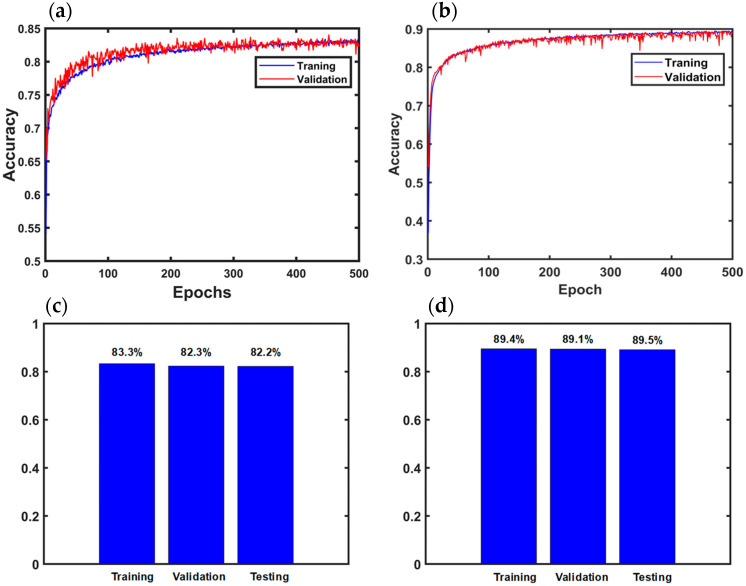
(**a**) The learning curve of the neural network model for cotton type classification using XBO lamp data, illustrating the model’s performance, as evaluated on both training and validation datasets across each epoch of the training process. (**b**) The learning curve of the neural network model for cotton type classification using deuterium lamp data, illustrating the model’s performance, as evaluated on both training and validation datasets across each epoch of the training process. Model accuracies achieved after the final training epoch for the training, validation, and test datasets using (**c**) deuterium lamp and (**d**) XBO lamp.

Table 3 presents the classification results for the XBO lamp. The model achieved an accuracy of 83.6% and a sensitivity of 84%. Table 4 shows the classification matrix for the deuterium lamp, where the accuracy was 90.1% and the sensitivity was 89.5%. Additionally, the learning curves of the neural network for cotton type classification using the deuterium and XBO data, along with the model accuracies reached after the final training epoch for the training, validation, and test datasets, are presented in Appendix A Figure A5.

Table 3 reports that 7402 spectra were collected with the XBO light source, while Table 4 shows that 9038 spectra were obtained using the deuterium light source. The number of spectra shown in Table 1, Table 2, Table 3 and Table 4 differed slightly due to the manual selection and positioning of the samples. Additionally, some outlier spectra were removed. The results from the hyperspectral imaging using an XBO light source demonstrate that the HP spectra were almost correctly classified as distinct from those of cotton. However, there were misclassifications: 302 MCC spectra were predicted as StC, and 132 StC spectra were predicted as MCC. Many RcBC spectra were misclassified as StC, supporting the hypothesis that the RcBC could have been recycled from conventional cotton. Some OC spectra were misclassified as RcBC and StC, which indicates their high similarity to each other. Nearly 100% of the RcC spectra were correctly classified and distinguished. The results from the hyperspectral imaging using the deuterium light source demonstrate that the spectra of conventional cotton (StC) were accurately classified as distinct from those of HP and RcC, with only minor misclassifications: 30 OC spectra were predicted as StC, and 78 StC spectra were predicted as OC. Additionally, 72 RcBC spectra were misclassified as StC, further supporting the hypothesis that the RcBC may have been recycled from conventional cotton. One MCC spectrum was misclassified as OC, fourteen MCC spectra were misclassified as RcBC, and thirty MCC spectra were misclassified as StC, indicating a high similarity among these samples. Nearly 100% of the RcC spectra and 98% of the HP spectra were correctly classified and distinguished. In conclusion, conventional cotton could be distinguished from organic cotton, recycled cotton could be distinguished from raw cotton, and hemp plant could be distinguished from cotton. As a result, the classifications achieved through deep learning were largely comparable to those obtained using the simpler PCA-QDA method.

**Figure 5:**
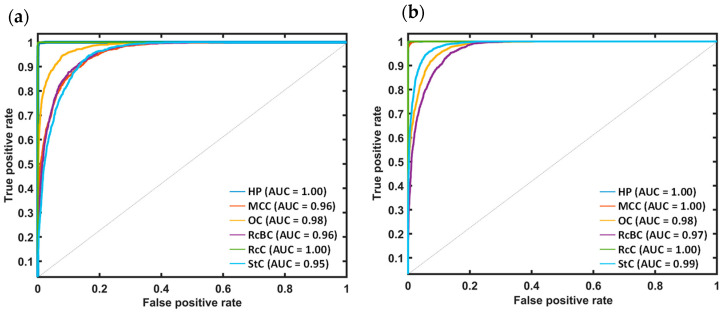
ROC curve and corresponding AUC values for the developed neural networks to classify different types of cotton using (**a**) XBO and (**b**) deuterium light sources. The calculation was performed using the one-vs-rest method for multiclass classification problems.

A conventional analysis was employed to validate the neural network models. The receiver operating characteristics curve (ROC) and corresponding area under the curve (AUC) were calculated to assess the robustness of the developed neural networks. Figure 5 shows the ROC curve and corresponding AUC values using the XBO and deuterium light sources. The XBO and deuterium light source results demonstrate that the HP and RcC were 100% correctly classified from the other cotton types. Furthermore, 100% discrimination of the MCC data was achievable with the deuterium lamp, compared to 96% with the XBO lamp. The OC samples are classified with 98% for both models. Additionally, RcBC and StC reached 96% and 95% using the XBO lamp and 97% and 99% utilizing the deuterium light source. In conclusion, these two models were highly robust regarding the different classification cutoffs. Overall, the model’s predictive performance using the deuterium lamp was slightly better than that using the xenon lamp, although both models performed comparably well.

It is noted that there was some overlap between the samples in the models, which was anticipated given the diversity of the cotton types studied. Despite the inherent differences in these natural materials, they exhibit substantial similarities in their properties, posing a significant challenge. Al Ktash et al. [27] successfully distinguished between different types of cotton using hyperspectral imaging in the NIR region, with results derived from complex spectral preprocessing techniques. In contrast, this study focused on the UV region, which is more challenging for many natural products, yet it successfully differentiated between the various cotton types using a more straightforward preprocessing method. Building on the findings from previous work with a laboratory spectrometer [3,18,42,43], this study demonstrates that the sample set could be successfully analyzed in the UV wavelength range using hyperspectral imaging with XBO and deuterium light sources, achieving comparable results. The main differences between the studies are shown in Appendix A, Table A1.

The observed differences in the spectral characteristics and classification accuracy between the two light sources can be primarily attributed to their respective spectral outputs and the corresponding absorbance properties of the analyzed materials. The XBO lamp’s broad spectrum facilitates the detection of a wide array of molecular signatures but may introduce overlapping signals among different cotton types. Conversely, the deuterium lamp’s focused UV output enhances sensitivity and resolution, allowing for a more precise identification of specific biomolecules associated with cotton types. This underscores the importance of selecting appropriate light sources for optimizing spectral analysis in material classification [16,34,35,36,37].

### 3.2. Influence of Different Light Sources

**Figure 6:**
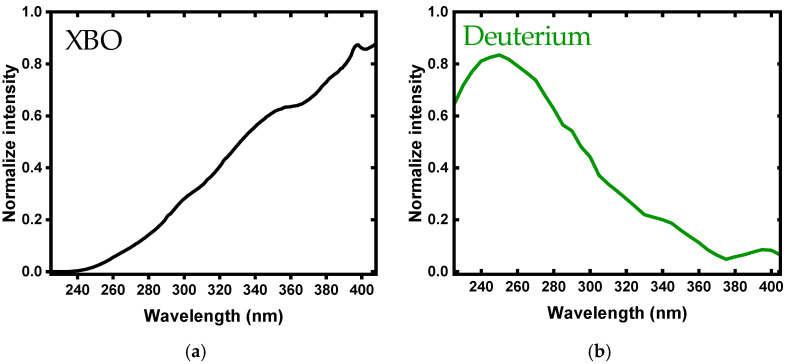
Normalized intensity of (**a**) XBO and (**b**) deuterium lamps [16].

Figure 6 shows the normalized intensity of the XBO and deuterium lamps. Figure 6a shows that the intensity of the XBO lamp in the UVC range was nearly zero and began to increase gradually. In contrast, as shown in Figure 3b, the intensity of the deuterium lamp was high in the deep UV range and continued through to the UVA range. The choice of light source significantly impacts the spectral characteristics and analysis outcomes in hyperspectral imaging. This study utilized two distinct light sources, namely, an XBO lamp and a deuterium lamp, each offering unique spectral properties. The XBO lamp produces a broad spectrum of light, covering the UV region of 225–408 nm [16,34,36,37]. The averaged spectra obtained using the XBO light source revealed distinct features, with prominent bands at <250 nm, 260 nm, 280 nm, 310 nm, and 375 nm, corresponding to key biomolecules such as proteins and DNA. This enabled differentiation between the various cotton types, including between organic and conventional cotton. In contrast, the deuterium lamp is specifically designed for UV applications, emitting light primarily in the UV range of 160–400 nm [34,35,36]. The spectral features observed with the deuterium lamp highlighted bands around 230 nm, 261 nm, and 307 nm, crucial for identifying the same biomolecules but with different intensity patterns. Notably, the deuterium lamp exhibited clearer intensity contrasts, especially in the range from 236 nm to 366 nm, enhancing the model’s ability to distinguish between the cotton types, particularly between organic and recycled cotton. The influence of the light source is further evident in the PCA results, where the deuterium lamp achieved an overall classification accuracy of 85.1% compared to 76.1% for the XBO lamp. This difference in classification performance indicates that the spectral characteristics provided by the deuterium lamp allowed for more precise discrimination among the cotton types. Thus, while both light sources could effectively distinguish between the different cotton types, the deuterium lamp’s enhanced sensitivity and clearer spectral contrasts make it the preferred choice for applications focused on UV spectral analysis.

## 4. Conclusions

This study demonstrates the effectiveness of UV hyperspectral imaging combined with PCA for classifying and analyzing different cotton types and hemp samples. By utilizing hyperspectral imaging with two different light sources, namely, xenon arc (XBO) and deuterium lamps in the UV range (225–408 nm), this study successfully captured the detailed spectral profiles of various raw cotton samples, including organic raw cotton (OC), hemp plant (HP), recycled cotton (RcC), standard raw cotton (StC), recycled organic bright cotton (RcBC), and mechanically cleaned cotton (MCC).

PCA analysis revealed that the first three PCs accounted for approximately 94.8% of the total variance when using the XBO light source and 89.4% with the deuterium light source, enabling the distinction between the different cotton types and hemp samples. This study also found that combining PCA with QDA further improved the classification accuracy, achieving an overall accuracy of 76.1% for the XBO light source and 85.1% for the deuterium light source for the cotton types. These results allow for the differentiation between conventional cotton (StC) and organic cotton (OC), as well as between hemp (HP) and all cotton types. Furthermore, this method of analysis can distinguish between different types of recycled cotton (RcC and RcBC), indicating variations in recycling processes across companies. The recycled cotton (RcBC) closely resembled the raw cotton (StC), suggesting that it may have been derived from the same original source. A fully connected neural network, a deep learning technique for classification, was also employed. The classification accuracy achieved for the XBO light source was 83.6%, while the deuterium light source yielded an accuracy of 90.1%.

These findings indicate that hyperspectral imaging in the UV range and advanced data analysis techniques provide a powerful tool for quality control and classification in textile manufacturing. This method offers a valuable alternative to traditional off-line techniques, addressing their limitations and enabling a more comprehensive analysis of cotton properties. Overall, this study supports the integration of UV hyperspectral imaging into the textile industry, particularly for raw cotton classification and quality assessment. This advancement enhances material analysis, contributing to improved quality control and economic efficiency in cotton production.

## Figures and Tables

**Table 1:** Confusion matrix of the PCA-QDA model with five PCs projected (overall accuracy of 76.1%) using XBO light source.

Predicted							
Reference		HP	MCC	OC	RcBC	RcC	StC
	HP	961	3	56	0	0	0
	MCC	0	664	48	79	2	227
	OC	42	28	838	97	11	4
	RcBC	0	174	70	567	4	205
	RcC	0	1	0	0	1018	1
	StC	0	104	68	241	1	606

**Table 2:** Confusion matrix of the PCA-QDA model with five PCs projected (overall accuracy of 85.1%) using deuterium light source.

Predicted							
Reference		HP	MCC	OC	RcBC	RcC	StC
	HP	963	0	11	0	0	0
	MCC	0	793	37	47	0	25
	OC	7	132	582	223	1	26
	RcBC	4	46	71	709	0	87
	RcC	0	0	0	0	953	0
	StC	0	19	33	82	0	845

**Table 3:** Confusion matrix of the fully connected neural network (overall accuracy of 83.6%) using XBO light source.

Predicted							
Reference		HP	MCC	OC	RcBC	RcC	StC
	HP	961	0	4	5	0	0
	MCC	2	839	48	78	0	302
	OC	6	23	1152	154	1	55
	RcBC	1	69	80	942	3	128
	RcC	0	0	2	1	1133	0
	StC	0	132	51	117	0	1117

**Table 4:** Confusion matrix of the fully connected neural network (overall accuracy of 90.1%) using deuterium light source.

Predicted							
Reference		HP	MCC	OC	RcBC	RcC	StC
	HP	1543	0	9	0	0	0
	MCC	0	1151	1	14	0	4
	OC	4	1	1220	331	6	30
	RcBC	0	12	119	1344	5	72
	RcC	0	0	1	0	1549	0
	StC	0	17	78	294	2	1231

## Data Availability

The data presented in this study are available on request from the corresponding author due to the massive data volume.

## Appendix A

### Appendix A.3. Neural Network Using XBO and Deuterium Lamps

The learning curve of the neural network model for cotton type classification using the data generated from both lamps illustrates the model’s performance, as evaluated on both the training and validation datasets across each epoch of the training process, Figure A5a,b. Model accuracies achieved after the final training epoch for the training, validation, and test datasets, Figure A5c,d.

The training dataset was used to calculate the model, and the validation set was used to tune the model’s hyperparameters during training. Finally, the test set was used to evaluate the model’s performance on a fully independent dataset.

**Table A1:** Comparison between the results of UV and NIR hyperspectral imaging for distinguishing between various cotton types and hemp samples.

Aspect	UV Hyperspectral Imaging		NIR Hyperspectral Imaging [27]
	XBO Lamp	Deuterium Lamp	
Spectral range/nm	225–408	225–408	1100–2200
Number of PCs used	3	3	3
PCA variance explained (3 PCs)	94.8%	89.4%	88.2%
Overall accuracy (PCA-QDA)	76.1%	83.1%	Not provided
Data pre-processing	Simple	Simple	Complex
Prototype	Expensive	Expensive	Cheap
